# Machine learning for outcome prediction in patients with non-valvular atrial fibrillation from the GLORIA-AF registry

**DOI:** 10.1038/s41598-024-78120-z

**Published:** 2024-11-07

**Authors:** Martha Joddrell, Wahbi El-Bouri, Stephanie L. Harrison, Menno V. Huisman, Gregory Y. H. Lip, Yalin Zheng, GLORIA-AFinvestigators

**Affiliations:** 1 Liverpool Centre for Cardiovascular Science at University of Liverpool, Liverpool John Moores University and Liverpool Heart and Chest Hospital, Liverpool, UK; 2 Department of Cardiovascular and Metabolic Medicine, Institute of Life Course and Medical Sciences, University of Liverpool, William Henry Duncan Building, 6 West Derby St, Liverpool, L7 8TX UK; 3 Department of Medicine – Thrombosis and Hemostasis, Leiden University Medical Center, Leiden, The Netherlands; 4 Department of Eye and Vision Sciences, University of Liverpool, Liverpool, UK; 5 Department of Clinical Medicine, Danish Center for Clinical Health Services Research, Aalborg University, Aalborg, Denmark

**Keywords:** Interventional cardiology, Mathematics and computing

## Abstract

Clinical risk scores that predict outcomes in patients with atrial fibrillation (AF) have modest predictive value. Machine learning (ML) may achieve greater results when predicting adverse outcomes in patients with recently diagnosed AF. Several ML models were tested and compared with current clinical risk scores on a cohort of 26,183 patients (mean age 70.13 (standard deviation 10.13); 44.8% female) with non-valvular AF. Inputted into the ML models were 23 demographic variables alongside comorbidities and current treatments. For one-year stroke prediction, ML achieved an area under the curve (AUC) of 0.653 (95% confidence interval 0.576–0.730), compared to the CHADS_2_ and CHA_2_DS_2_-VASc scores performance of 0.587 (95% CI 0.559–0.615) and 0.535 (95% CI 0.521–0.550), respectively. Using ML for one-year major bleed prediction increased the AUC from 0.537 (95% CI 0.518–0.557) generated by the HAS-BLED score to 0.677 (95% CI 0.619–0.724). ML was able to predict one-year and three-year all-cause mortality with an AUC of 0.734 (95% CI 0.696–0.771) and 0.742 (95% CI 0.718–0.766). In this study a significant improvement in performance was observed when transitioning from clinical risk scores to machine learning-based approaches across all applications tested. Obtaining precise prediction tools is desirable for increased interventions to reduce event rates.

*Trial Registry*https://www.clinicaltrials.gov; Unique identifier: NCT01468701, NCT01671007, NCT01937377.

**Supplementary Information:**

The online version contains supplementary material available at 10.1038/s41598-024-78120-z.

## Introduction

Risk stratification scores are used to determine the likelihood of an outcome occurring, guiding appropriate treatment and therapy interventions. Existing methods for stroke and major bleeding prediction in patients with atrial fibrillation (AF) are typically developed using traditional statistical approaches, hence, in many cases under-perform and are “oversimplified”^1^. One study demonstrated that four commonly-used cardiovascular risk stratification tools overestimated the intended outcome risk by 8-67% in women and 37-154% in men^2^.

Clinical scores are often designed to be easy-to-use and make fast predictions without the need for extensive training. However, these scores are frequently created with strict requirements including stringent feature composition that limit predictive ability^3^. Lack of generalisation of these tools results from their development on outdated cohorts. With life expectancy increasing and treatment options progressing, patients today have different factors contributing to disease development, with this change known as `data shift’^4^.

AF is the most frequent arrhythmia worldwide^5^, and is often asymptomatic but carries an increased mortality and morbidity risk^6^. To reduce the risk of stroke, AF patients are usually recommended oral anticoagulation, which needs to be balanced against a potential increase in bleeding likelihood^7,8^.

Approaches exploring improvement of current risk scores have begun exploiting machine learning (ML), which embeds greater complexity and interactions of information. Recent studies have shown improvement through ML when predicting AF^9,10^ with subsequent validation using an external data^11^, predicting the risk of developing AF post-stroke^12^, and predicting outcomes in individuals with AF, including numerous studies on stroke prediction^13^.

In contrast, some have reported that ML does not always improve prediction. A 2022 review concluded a limited indication that ML can move beyond classical scores or basic logistic regression when attempting to predict a specific AF-associated risk^14^. Auxiliary analyses showed that ML did not achieve superior performance over clinical scores when predicting stroke, major bleeding, or mortality on two AF-based registries^15^. Therefore, further studies are needed to confirm the appropriateness, applicability, and performance on a range of diverse datasets with associated and extensive model validation.

In the present study, validated clinical risk scores for stroke and major bleeding are compared with ML-based approaches that incorporate a more complex formulation between a larger set of features to assess absolute difference in approaches. Comparative performance is deemed in terms of area under the curve (AUC), sensitivity, and specificity, with optimal models selected by highest AUC value. Prediction of all-cause mortality using ML models are also reported.

## Methods

Many studies provide a comprehensive overview of current clinical risk scores for AF-associated outcomes^16,17^. Recommendations for standard reporting^18,19^ are followed to ensure transparency, including a critical appraisal’s suggestions for cardiovascular artificial intelligence (AI) applications^20^.

### Study population

**Fig. 1:**
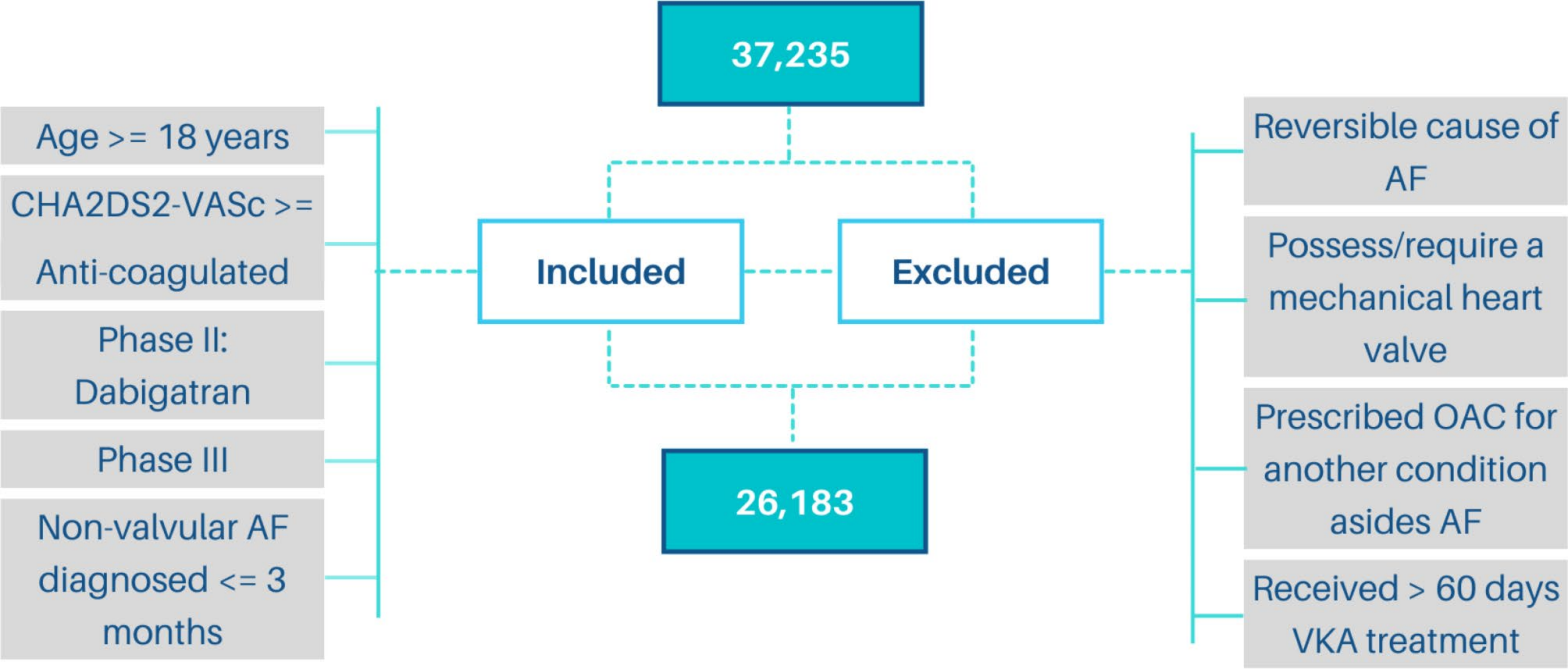
Patient inclusion criteria resulting in 26,183 patients.

The GLORIA-AF registry^21^ is an observational, prospective cohort of 37,235 patients with recently diagnosed non-valvular AF at risk of a stroke. Data were collected between 2011 and 2020 from a global registry program located in over 600 locations within 21 countries. For the purpose of this analysis, not all patients were included. Material S1 details the inclusion and exclusion criteria. Figure 1 demonstrates the final cohort of 26,183 used for this analysis. As the GLORIA-AF registry contains hundreds of variables, for this analysis 23 patient history, demographic, comorbidities, and medication variables were manually selected based on clinical expertise. All 23 variables (Table 1) were included in the model.

**Table 1:** Variable characteristics that will be included in the models, along with the associated mean, standard deviation, and percentage of the variable that contains missing data.

Baseline characteristics	Total cohort (% population)	Mean (SD)	Missing (% population)
Age (years) 18–44 45–54 55–64 65–74 75–90	423 (1.6) 1549 (6.0) 4769 (18.2) 9485 (36.2) 9541 (36.4)	70.13 (10.13)	416 (1.6)
Gender Female Male	11,733 (44.8) 14,450 (55.2)		0 (0.0)
Heart rate		80.1 (21.3)	210 (0.8)
Systolic blood pressure		132.2 (18.7)	196 (0.8)
Diastolic blood pressure		78 (12)	199 (0.8)
Height (cm)		168.2 (10.3)	265 (1.0)
Weight (kg)		81.3 (20.6)	219 (0.8)
BMI		28.6 (6.3)	285 (1.0)
Treatment Group Apixaban ASA Dabigatran Edoxaban Rivaroxaban VKA Antiplatelets other than ASA None	4505 (17.2) 2163 (8.3) 8722 (33.3) 332 (1.3) 4015 (15.3) 4836 (18.5) 213 (0.8) 1386 (22.8)		11 (0.04)
Region Africa/Middle East Asia Europe Latin America North America	314 (1.2) 4905 (18.7) 12,993 (49.6) 2007 (7.7) 5964 (22.8)		0 (0.0)
Race Arab/Middle East Asian Black/Afro-Caribbean White Other	331 (1.3) 4557 (17.4) 453 (1.7) 18,200 (69.5) 895 (3.4)		1747 (6.7)
Alcohol use No alcohol Less than 1 drink/week 1–7 drinks/week More than 7 drinks/week	11,411 (43.6) 6312 (24.1) 5094 (19.4) 1770 (6.8)		1596 (6.1)
Smoking status Never smoked Current smoker Ex-smoker	15,155 (57.9) 2443 (9.3) 7750 (29.6)		835 (3.2)
Types of AF			0 (0.0)
Paroxysmal	14,543 (55.5)		
Persistent	9000 (34.4)		
Permanent	2649 (10.1)		
Hypertension	19,671 (75.7)		56 (0.2)
Diabetes mellitus	6067 (23.2)		
Coronary artery disease	4932 (18.8)		662 (2.5)
Peripheral artery disease	756 (2.9)		196 (0.7)
Previous thromboembolism	3889 (14.9)		0 (0.0)
Previous myocardial infarction	2492 (9.5)		18 (0.07)
Previous deep vein thrombosis	303 (1.2)		315 (1.2)
Complex aortic plaque	225 (1.0)		5938 (22.7)
Respiratory disease	2923 (11.2)		270 (1.0)
Outcomes			
Major bleed	873 (3.3)		0 (0.0)
Stroke	681 (2.6)		0 (0.0)
All-cause death	2328 (8.9)		0 (0.0)

GLORIA-AF is a global registry, and the study was approved by local institutional review boards at each participating centre. There were multiple participating centres, in which the protocol was approved; these are listed in ClinicalTrials.gov (NCT01468701, NCT01671007, and NCT019373770). All participants provided informed consent. All studies were performed in accordance with the Declaration of Helsinki.

## Outcomes

Three outcomes are predicted in the subsequent analysis; stroke, major bleeding, and all-cause mortality. Characterisation of these outcomes is provided in Material S2.

## Data pre-processing

Data were initially split by a 70:30 ratio into training and testing sets before further pre-processing on the training cohort. Multiple imputation (m = 5, maxit = 50) was performed to avoid a substantial reduction in sample size. Random Over-Sampling Examples (ROSE) was implemented to counteract the outcome variable’s large class imbalance. More detail on both multiple imputation and ROSE method is provided in Material S3. Normalisation was applied to numerical variables and one-hot encoding to categorical variables with more than two levels.

## Current clinical risk scores for stroke and major bleeding

The CHADS_2_score was developed to estimate the risk of stroke in AF patients^22^. A total of 6 points can be obtained (**C**ongestive heart failure + 1, **H**ypertension + 1, **A**ge $\:\ge\:$ 75 + 1, **D**iabetes mellitus + 1, previous **S**troke/TIA history + 2) via this score. Patients with a score of $\:\ge\:$2 are generally considered for anti-coagulation. The CHA_2_DS_2_-VASc score^8^, also calculates the risk of stroke; included are the same risk factors except for: **A**ge +1 when 65–74 and +2 $\:\ge\:$ 75, **V**ascular disease +1 (including prior myocardial infraction, peripheral artery disease, or aortic plaque), and **S**ex: female +1. Here, a total of 9 points can be summed; again, oral anti-coagulation is recommended for anyone with a score of 2 or more.

The HAS-BLED Score^23^ is a 9 point-based score used to determine the risk of major bleeding in patients prescribed anticoagulation. Within this score, a summation of the risk factors includes: **H**ypertension + 1, **A**bnormal renal or liver function + 1–2, **S**troke history + 1, **B**leeding predisposition or tendency + 1, **L**abile INR + 1, Age $\:\ge\:$ 65 (**E**lderly) +1, and **D**rugs (for bleeding predisposition such as aspirin or NSAIDs) or alcohol + 1–2.

When calculating the performance of clinical risk scores, any patient classified as high risk by these scores within the specified study period would receive a prediction of stroke. Although the moderate classes still carry a risk of stroke, for the purpose of this analysis they were assigned ‘no stroke’ which allows insight into how the score performs under certain conditions - else, ~ 100% of patients would have a positive prediction if the combination of moderate- and high-risk was used.

## Machine learning approaches

ML classification models included logistic regression (LR)^24^, random forest (RF)^25^, linear discriminant analysis (LDA)^26^, naive Bayes (NB)^27^, eXtreme Gradient Boosting (XGB)^28^, and neural network (NN)^29^.

All models were trained with 10-fold cross validation. For appropriate comparison with the clinical risk scores, both 3-year (total dataset) and 1-year (event within 12 months) cohorts were tested. The models were assessed using AUC, sensitivity, and specificity but performance was primarily determined based on the AUC. DeLong’s statistical significance test for comparing AUCs was used to differentiate improvements between methods at the 95th percentile. AUC confidence intervals at the 95% level were also reported. Analyses were conducted using python and R. More information on model development is provided in Material S4.

## Results

### Population characteristics

The mean age of the population was 70.13 (standard deviation (SD) 10.13) and 44.8% were female. Table 1 provides further detail on the overall population. Within the supplementary material, the population characteristics of those who had the outcome of stroke (Material S7) and major bleeding (Material S8) is provided. All 23 variables (not including the outcomes) in Table 1 were used to build the ML models.

## Clinical risk scores

**Fig. 2:**
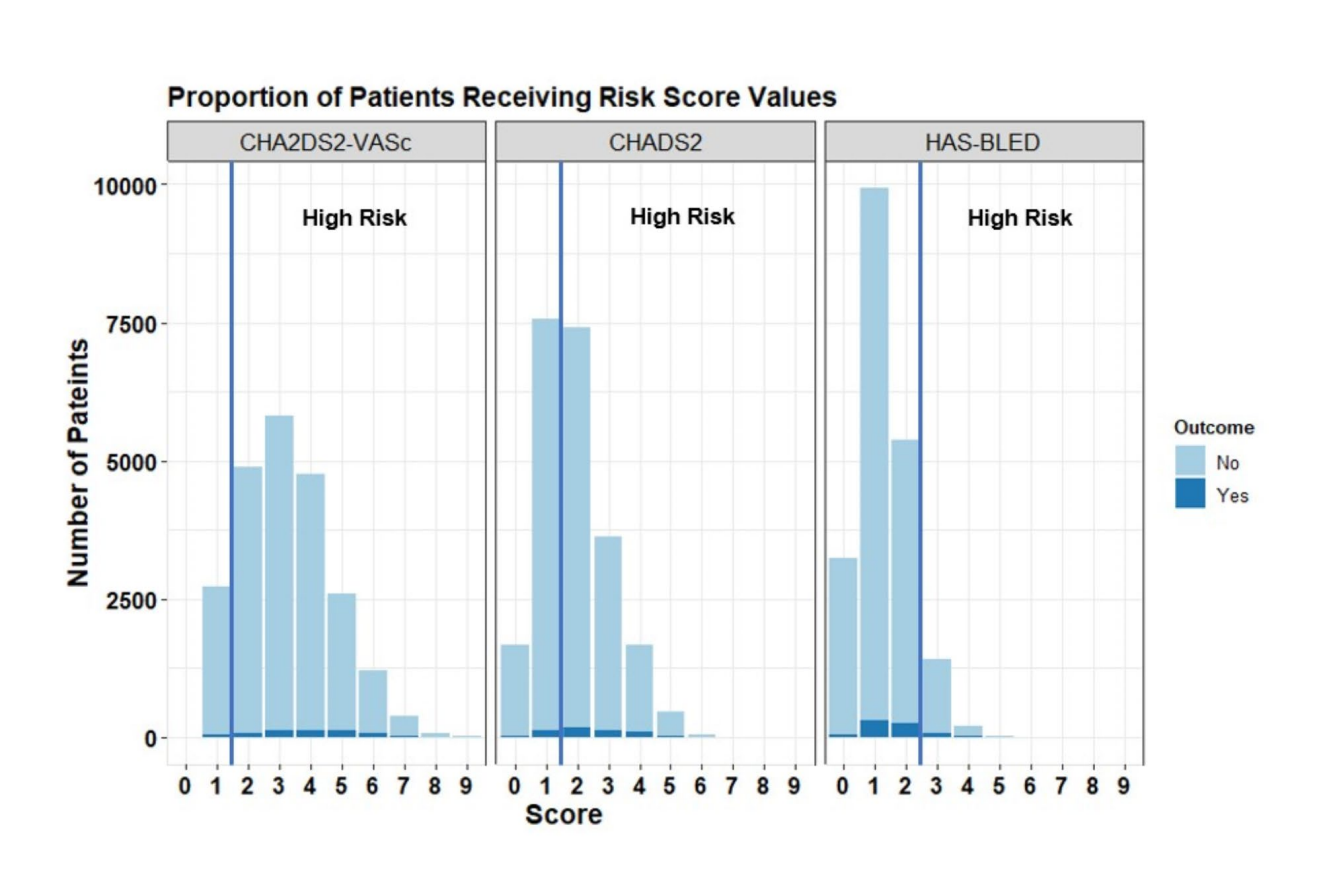
Bar plot displaying the proportion of patients receiving moderate/high risk outcomes from the clinical risk scores, stratified by the proportion that had the outcome.

Of those with complete data (*n* = 26,183), 681 (2.6%) patients had an outcome of stroke over the 3-year study period. However, 22,520 patients (86%) had been classified as high risk by the CHA_2_DS_2_-VASc score (score $\:\ge\:$ 2). Contrasting, 14,994 (57.3%) patients were deemed high risk (score $\:\ge\:$ 2) by the CHADS_2_ score. Over the total study period, the number of patients who experienced a major bleed was 873 (3.3%). Consequently, the HAS-BLED score deemed 2,323 (8.9%) patients as high risk (score $\:\ge\:$ 3). Table 2 displays the number of patients obtaining each level of these current clinical risk scores. The mean (SD) of the CHADS_2_, CHA_2_DS_2_-VASc, and HAS-BLED scores were 1.9 (1.2), 3.2 (1.5), and 1.4 (0.9) respectively. Figure 2 demonstrates the complexity of predicting events using these simple risk categorisation methods.

**Table 2:** Performance metrics of the CHADS_2_, CHA_2_DS_2_-VASc, and HAS-BLED clinical risk scores.

	Sensitivity	Specificity	AUC	95% CI (AUC)
1-year CHADS CHA_2_DS_2_-VASc HAS-BLED	0.761 0.948 0.155	0.413 0.123 0.92	0.587 0.535 0.537	(0.559, 0.615) (0.521, 0.55) (0.518, 0.557)
3-year CHADS_2_ CHA_2_DS_2_-VASc HAS-BLED	0.738 0.933 0.125	0.415 0.123 0.921	0.577 0.528 0.522	(0.557, 0.6) (0.517, 0.54) (0.51, 0.535)

The 1- and 3-year AUCs calculated for stroke risk by the CHADS_2_ score were 0.587 (95% CI 0.559–0.615) and 0.577 (95% CI 0.557-0.600), respectively and for the CHA_2_DS_2_-VASc score, 0.535 (95% CI 0.521–0.550) and 0.528 (95% CI 0.517–0.540). When assessing 1-year stroke prediction as the two scores were designed for, the CHADS_2_ risk score was able to correctly classify 0.761 of actual strokes (recall), however it only achieved a precision value of 0.013 as it over-predicted the number of patients who would have a stroke, classifying almost half of the patients as stroke (50.4%) when in reality this value was much lower (0.026). Additionally, it obtained a 1-year/3-year sensitivity of 0.761/0.738 and specificity of 0.413/0.415. CHA_2_DS_2_-VASc performed similarly with a recall of 0.948 and precision value of 0.011. However, this score classified a significantly larger number of patients as predicted stroke (75.2%) when in reality the true rate of stroke in the observed study duration was 2.6%. The associated sensitivities and specificities for 1-year/3-year prediction were 0.948/0.933 and 0.123/0.123, respectively.

When estimating major bleeding risk, the HAS-BLED score obtained a 1-year AUC of 0.537 (95% CI 0.518-557), decreasing marginally to 0.522 (95% CI 0.510–0.535) for the 3-year cohort. The HAS-BLED score had a specificity of 0.921, compared with a sensitivity value of 0.125 for 3-year prediction, representing a very low rate of outcome detection. Similarly, at 1-year prediction the sensitivity and specificity values achieved were 0.155 and 0.920, respectively. The precision of this score for 1- and 3-year prediction was reported at 0.030 and 0.054, respectively, demonstrating over-prediction of the outcome compared with the true event rate.

**Fig. 3:**
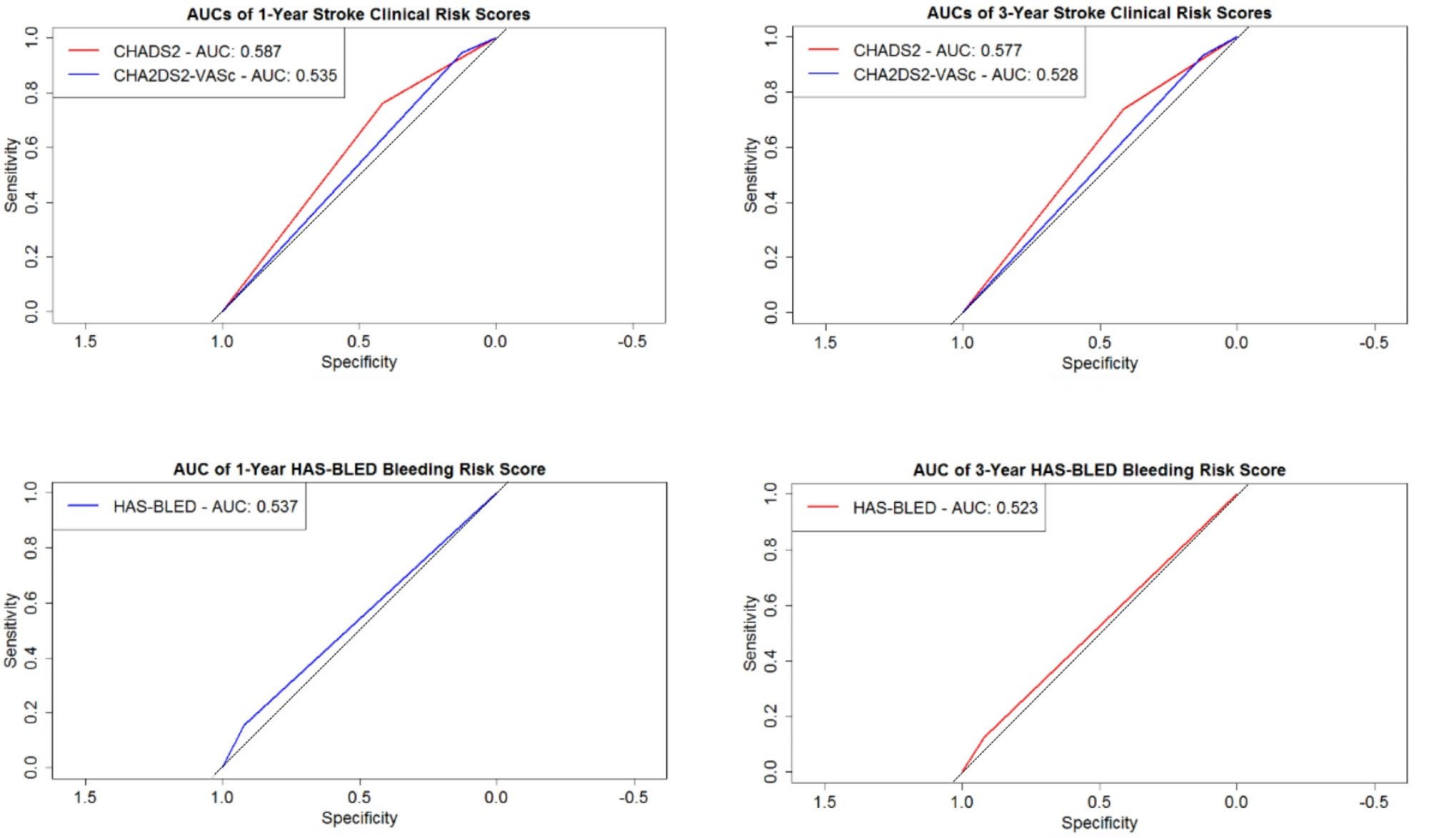
1-year and 3-year clinical risk score (CHADS_2_, CHA_2_DS_2_-VASc, and HAS-BLED) receiver operating characteristic (ROC) curves for the prediction of stroke and major bleeding.

Performance of the CHADS_2_ and CHA_2_DS_2_-VASc scores for 1-year stroke risk was similar to 3-year stroke risk (AUCs CHADS_2_ 1-/3-year: 0.587/0.577, p-value 0.547 and AUCs CHA_2_DS_2_-VASc 1-/3-year: 0.535/0.528, p-value 0.452). No statistically significant difference was observed between the AUCs of 1- and 3-year prediction of major bleeding (p-value 0.215; AUC 1-year 0.537, 3-year 0.522). Figure 3 displays the ROC curves for the clinical risk scores. Subsequently, it was tested to determine if altering the numerical threshold categorising patients into ‘stroke’/‘no stroke’ had any effect on performance of the methods (Materials S5).

### Machine learning models

**Fig. 4:**
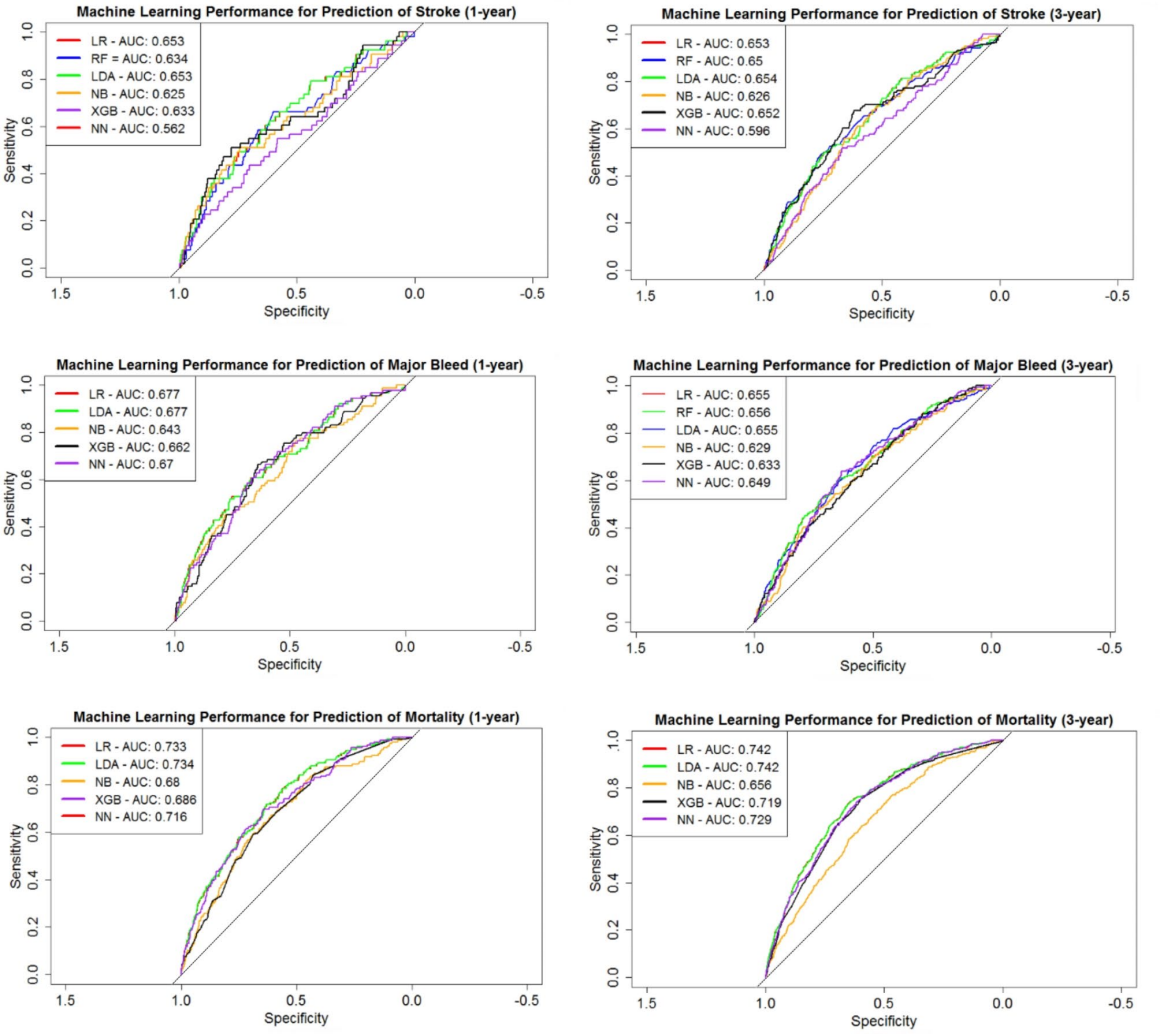
1-year and 3-year machine learning ROCs for prediction of stroke, major bleed, and mortality.

Figure 4 displays the ROC curves for all models. When determining performance, it is important to consider the threshold boundary for classification. In practice, sensitivity or specificity will be optimized to reflect the consequences and clinical priorities of an application. Material S6 provides a more detailed explanation of the need for a trade-off.

Table 3 reports the metrics when sensitivity and specificity have been balanced by thresholding for each model. Beyond that, the thresholds have been adjusted to maximise sensitivity whilst attempting to keep the specificity at an acceptable rate (> 50%), to increase the degree of events captured. Typically, the Youden Index is used to determine the appropriate threshold, however it attempts to balance both sensitivity and specificity, which for this application is not ideal. Table 4 gives the performance of the machine learning models maximised for either sensitivity or specificity.

**Table 3:** Performance of 1-year and 3-year ML models for the prediction of stroke, major bleed, and all-cause mortality.

	Balanced		Optimised		AUC	95% CI AUC
	Sensitivity	Specificity	Sensitivity	Specificity		
1-year stroke						
Logistic regression	0.623	0.617	0.698	0.530	**0.653**	(0.576, 0.73)
Random forest	0.623	0.615	0.660	0.602	0.634	(0.556, 0.712)
Linear discriminant analysis	0.623	0.615	0.698	0.528	**0.653**	(0.577, 0.73)
Naïve Bayes	0.604	0.561	0.642	0.542	0.625	(0.541, 0.709)
XGBoost	0.604	0.565	0.642	0.527	0.633	(0.549, 0.717)
Neural network	0.566	0.533	0.566	0.533	0.562	(0.478, 0.646)
3-year stroke						
Logistic regression	0.602	0.594	0.720	0.504	0.653	(0.603, 0.704)
Random forest	0.627	0.604	0.695	0.519	0.650	(0.597, 0.703)
Linear discriminant analysis	0.602	0.594	0.720	0.504	**0.654**	(0.604, 0.704)
Naïve Bayes	0.610	0.606	0.703	0.513	0.626	(0.578, 0.674)
XGBoost	0.636	0.633	0.703	0.573	0.652	(0.599, 0.705)
Neural network	0.576	0.554	0.619	0.501	0.596	(0.544, 0.649)
1-year major bleed						
Logistic regression	0.618	0.616	0.708	0.522	**0.677**	(0.62, 0.735)
Linear discriminant analysis	0.629	0.614	0.708	0.524	**0.677**	(0.619, 0.734)
Naïve Bayes	0.607	0.565	0.719	0.506	0.643	(0.583, 0.703)
XGBoost	0.652	0.641	0.753	0.527	0.662	(0.605, 0.719)
Neural network	0.640	0.631	0.742	0.506	0.670	(0.615, 0.724)
3-year major bleed						
Logistic regression	0.621	0.607	0.703	0.505	0.655	(0.616, 0.695)
Random forest	0.621	0.610	0.736	0.503	**0.656**	(0.616, 0.696)
Linear discriminant analysis	0.621	0.610	0.703	0.501	0.655	(0.616, 0.695)
Naïve Bayes	0.593	0.590	0.698	0.500	0.629	(0.589, 0.669)
XGBoost	0.599	0.587	0.670	0.501	0.633	(0.594, 0.672)
Neural network	0.637	0.632	0.714	0.507	0.649	(0.61, 0.688)
1-year mortality						
Logistic regression	0.667	0.650	0.818	0.510	0.733	(0.695, 0.771)
Linear discriminant analysis	0.660	0.657	0.818	0.511	**0.734**	(0.696, 0.771)
Naïve Bayes	0.648	0.626	0.755	0.501	0.680	(0.638, 0.721)
XGBoost	0.673	0.600	0.686	0.59	0.686	(0.647, 0.725)
Neural network	0.660	0.656	0.786	0.500	0.716	(0.677, 0.756)
3-year mortality						
Logistic regression	0.684	0.683	0.827	0.505	**0.742**	(0.719, 0.766)
Linear discriminant analysis	0.684	0.682	0.827	0.507	**0.742**	(0.719, 0.766)
Naïve Bayes	0.616	0.612	0.733	0.500	0.656	(0.63, 0.682)
XGBoost	0.689	0.647	0.754	0.598	0.719	(0.695, 0.744)
Neural network	0.674	0.668	0.817	0.504	0.729	(0.705, 0.753)

Left: balanced sensitivity and specificity, right: optimised sensitivity. Bold highlights the highest AUC of each application.

**Table 4:** Performance of 1-year and 3-year machine learning models for the prediction of stroke, major bleed, and all-cause mortality.

	~ 90% sensitivity		~ 80% specificity		AUC	95% CI AUC
	Sensitivity	Specificity	Sensitivity	Specificity		
1-year stroke						
Logistic regression	0.906	0.248	0.377	0.849	**0.653**	(0.576, 0.730)
Random forest	0.887	0.219	0.415	0.79	0.634	(0.556, 0.712)
Linear discriminant analysis	0.906	0.247	0.377	0.850	**0.653**	(0.577, 0.730)
Naïve Bayes	0.906	0.184	0.330	0.792	0.625	(0.541, 0.709)
XGBoost	0.905	0.233	0.263	0.811	0.633	(0.549, 0.717)
Neural network	0.887	0.157	0.302	0.809	0.562	(0.478, 0.646)
3-year stroke						
Logistic regression	0.907	0.348	0.398	0.806	0.653	(0.603, 0.704)
Random forest	0.881	0.204	0.415	0.797	0.650	(0.597, 0.703)
Linear discriminant analysis	0.907	0.249	0.415	0.799	**0.654**	(0.604, 0.704)
Naïve Bayes	0.907	0.213	0.331	0.800	0.626	(0.578, 0.674)
XGBoost	0.890	0.216	0.398	0.797	0.652	(0.599, 0.705)
Neural network	0.907	0.172	0.339	0.806	0.596	(0.544, 0.649)
1-year major bleed						
Logistic regression	0.910	0.290	0.438	0.805	**0.677**	(0.620, 0.735)
Linear discriminant analysis	0.898	0.303	0.438	0.805	**0.677**	(0.619, 0.734)
Naïve Bayes	0.910	0.187	0.404	0.812	0.643	(0.583, 0.703)
XGBoost	0.880	0.266	0.202	0.895	0.662	(0.605, 0.719)
Neural network	0.910	0.306	0.360	0.822	0.670	(0.615, 0.724)
3-year major bleed						
Logistic regression	0.901	0.268	0.423	0.802	0.655	(0.616. 0.695)
Random forest	0.896	0.236	0.346	0.813	**0.656**	(0.616, 0.695)
Linear discriminant analysis	0.901	0.271	0.423	0.800	0.655	(0.616, 0.695)
Naïve Bayes	0.890	0.226	0.385	0.800	0.629	(0.589, 0.669)
XGBoost	0.901	0.235	0.363	0.802	0.633	(0.594, 0.672)
Neural network	0.901	0.219	0.352	0.800	0.649	(0.610, 0.688)
1-year mortality						
Logistic regression	0.906	0.372	0.503	0.798	0.733	(0.695, 0.771)
Linear discriminant analysis	0.906	0.373	0.503	0.799	**0.734**	(0.696, 0.771)
Naïve Bayes	0.906	0.208	0.403	0.804	0.680	(0.638, 0.721)
XGBoost	0.912	0.308	0.365	0.814	0.686	(0.647, 0.725)
Neural network	0.906	0.315	0.484	0.804	0.716	(0.677, 0.756)
3-year mortality						
Logistic regression	0.902	0.370	0.522	0.800	**0.742**	(0.719, 0.766)
Linear discriminant analysis	0.902	0.370	0.518	0.800	**0.742**	(0.719, 0.766)
Naïve Bayes	0.902	0.280	0.370	0.802	0.656	(0.630, 0.682)
XGBoost	0.906	0.334	0.436	0.817	0.719	(0.695, 0.744)
Neural network	0.902	0.363	0.473	0.800	0.729	(0.705, 0.753)

Left ~ 90% sensitivity and ~ 80% specificity; right optimised sensitivity. Bold highlights the highest AUC of each application.

When predicting stroke across the total cohort (3-year), the greatest AUC of 0.654 (95% CI 0.604–0.704) was generated by linear discriminant analysis. Improvement over the CHADS_2_ and CHA_2_DS_2_-VASc scores, which obtained respective 1-year AUCs of 0.570 (95% CI 0.559–0.615) and 0.535 (95% CI 0.521–0.550) was achieved using logistic regression, giving an AUC of 0.653 (95% CI 0.576–0.730). Most influential towards prediction of stroke within 1-year was age, race, and previous thromboembolism; two of which are incorporated within the existing clinical risk scores. For 3-year stroke, age, systolic blood pressure, previous thromboembolism, and complex aortic plaque were found to be significant predictors.

Achieving significantly higher performance than the HAS-BLED score (1-year AUC 0.537 95% CI 0.518–0.557; 3-year AUC 0.522 95% CI 0.510–0.535), the best ML model for 1-year and 3-year prediction of major bleeding was found through linear discriminant analysis and random forests, respectively, with corresponding AUCs of 0.677 (95% CI 0.619–0.724) and 0.656 (95% CI 0.616–0.696). Due to the HAS-BLED score being heavily skewed towards specificity for this dataset, the optimised sensitivities found through machine learning greatly improved from 0.155 to 0.708 for 1-year prediction and 0.125 to 0.736 when predicting 3-year likelihood. For prediction of both 1- and 3-year major bleeding, the two most influential predictors were age and region.

All the best performing ML models for each application achieved a statistically significant improvement (p-value < 0.05) in AUC over their comparative clinical score, with the exception of the 1-year application of the CHADS_2_ score (p-value = 0.114).

Mortality prediction of 1- and 3-year resulted in AUCs of 0.734 (95% CI 0.696–0.771) and 0.742 (95% CI 0.719–0.766), respectively, both through linear discriminant analysis. Predictors influential to mortality across both 1- and 3-year tasks were age, diastolic blood pressure, treatment of dabigatran or VKA, and the presence of respiratory disease.

## Discussion

A statistically significant increase using ML was obtained through all 3-year models and over both the 1-year CHA_2_DS_2_-VASc score and HAS-BLED score. Due to the cohort being comprised of AF patients entailing a greater risk of stroke, scores such as CHA_2_DS_2_-VASc will reflect this, classifying almost all patients as high risk. Additionally, the developed ML techniques are similarly hindered in their ability to classify low-risk patients, despite achieving greater performance.

Although ML comes with increased methodology complexity, discovery of alternative influential variables not currently incorporated into existing methods has been identified. As ML can embed an intricate combination of more risk factors, it allows for greater reflection of everyone - which is advantageous in personalised medicine.

AI is generating superior performance for clinical risk scores; however, this paper shows that these models are not faultless in all populations. Conflicting debates still occur regarding ML performance, and it is vital that similar studies continue to highlight concerns such as robustness and appropriateness in mediocre-performing cohorts.

This study was conducted within the constraints of available data, however external validation is a crucial step in ensuring a model’s generalisability in future cohorts which is a future avenue for this work. Due to conflicting reports of ML performance in which discrepancy mostly stems from the varying appropriateness of datasets, be it: data size, quality, or relevance, additional applications should use divergent sources to solidify the debate. Since previous work has shown greater predictive ability of outcomes using ML, use of GLORIA-AF for external validation of is encouraged.

As GLORIA-AF is a global registry, it is advantageous for reducing the impact of bias, however the vast geographical span may limit learning trends that predict a certain outcome in a community. Given the number of outcomes for stroke and major bleeding is low in this population, it is unsurprising that performance is limited. An increase in performance when predicting all-cause mortality may stem from the larger proportion of reported outcomes - evidence for increased data collection to capture more positive cases. Additionally, a combination of GLORIA-AF with a larger database may yield greater calibration and superior results.

When working with clinical data, the presence of missing data is unavoidable. Although multiple imputation was suitable for this application, it may not match the assumptions of alternative implementations. Hence, for validation studies, the process of handling missing data will need to be re-evaluated.

A final limitation rests on the desideratum of these findings. One contribution of this study is the application to a differing population; those with newly-diagnosed AF who have predominately been anti-coagulated. Since anti-coagulation is known to reduce the risk of stroke and increase the risk of bleeding, the cohort may not accurately reflect true risk in populations not on anticoagulation or those with varied usage patterns. Facilitation on reducing their risk are uncertain and may require interventions such as dosage alteration. As the nature of the data containing primarily high-risk patients would have made a comparison of methods improbable, the choice to assign high-risk with an outcome ‘stroke’ and moderate-risk as ‘no stroke’ should be noted as a limitation when considering these findings.

AF patients at higher risk for embolic strokes are also at a higher risk for stroke due to atherosclerosis, but given the limitations of the GLORIA-AF data, it was not possible to fully ascertain whether these patients that had a stroke while on anticoagulants are actually those with a residual embolic risk despite anticoagulation, or those with a higher thrombotic risk.

Despite these limitations, there are numerous beneficial clinical implications of updating existing model’s including gaining a statistically significant increase in predictive performance, which can allow more timely interventions. Additionally, by embedding the most current and relevant data, tailored risk management can improve through individualised stratification. Models such as the ones in this study can be seamlessly integrated into clinical work flow and electronic health record (EHR) systems to enable real-time risk assessment.

Asides from building upon clinical risk scores, ML is now being embedded into mobile health (mHealth) applications to aid ‘real time’ patient care. Attributing to advances in cloud computing and accessibility to novel technologies, mHealth research and implementation is rapidly increasing. Through devices such as fitness watches, patients can be monitored continuously. Applications are also being implemented for diagnosis of AF^30^. Further development could transition into automated detection or prediction of changes in dynamic risk through time-series analysis. A recent review investigated mHealth apps relating to cardiovascular disease and reported on 38 studies for purposes including wearables for diagnosis^31^, emphasising this growing shift towards assistant automated healthcare.

## Conclusion

Current clinical risk scores for assessing the risk of stroke and major bleeding remain modest in performance hence spurring the development of more precise approaches. Machine learning has the potential to improve prediction by incorporating a more complex combination of risk factors. Any gain in outcome prediction will result in greater aversion of events but also reduce over-prescription to those without need of preventative treatment.

## Electronic supplementary material

Below is the link to the electronic supplementary material.

Supplementary Material 1

## Data Availability

The data that support the findings of this study are available from Boehringer Ingelheim but restrictions apply to the availability of these data, which were used under license for the current study, and so are not publicly available. Data are however available upon reasonable request and with permission of Boehringer Ingelheim (https://trials.boehringer-ingelheim.com/).
