# Supplementary material for: Machine learning for outcome prediction in patients with non-valvular atrial fibrillation from the GLORIA-AF registry
Source: Sci Rep. 2024 Nov 7;14:27088. doi: 10.1038/s41598-024-78120-z (PMC11544011; doi:10.1038/s41598-024-78120-z)
Supplement: Supplementary file 1 — Supplementary Material 1 [file 41598_2024_78120_MOESM1_ESM.docx]

**Supplemental Materials**

**Material S1.**

All phase III participants were selected but only those from phase II on dabigatran were included (due to the outcome of interest not being available for phase II participants on alternative treatments) who met the inclusion criteria ≥ 18 years, CHA2DS2-VASc score ≥ 1, and diagnosed with non-valvular AF within the previous 3 months. Participants were excluded if they received more than 60 days of prior Vitamin K antagonists (VKA) treatment, possess or require a mechanical heart valve, had a reversible AF cause, and had been prescribed oral anticoagulant (OAC) for conditions apart from AF. Only patients who had received treatment were used in the analyses for greater cohort distinction.

Material S2.

Stroke was characterised as the commencement, with assumed vascular origin, of a focal neurological deficit that lasted 24 hours or longer, or resulted in death. Stroke types included haemorrhagic, ischemic, or undetermined. Definition of major bleeding required at least one of the following: symptomatic bleeding in a vital organ/area: pericardial or intra-articular bleeding, retroperitoneal bleeding, intraspinal, intraocular, intracranial, or intramuscular along with compartment syndrome; overt bleeding with a decrease of at least 20 grams per litre in haemoglobin or resulting in at least 2 units transfused of blood or packed cells.

**Material S3.**

Multiple imputation is a technique that creates plausible estimates and datasets which are generated from a distribution based on the observed data along with variable relationships. Within the analysis, the R package ‘MICE (Multiple Imputation by Chained Equations)’ (Van Buuren, S., & Groothuis-Oudshoorn, K. (2011). mice: Multivariate imputation by chained equations in R. Journal of statistical software, 45, 1-67.) was used to generate 5 imputed datasets (m=5), by 50 iterations (maxit=50), with which the results were pooled to provide a final, complete dataset.

Random Over-Sampling Examples (ROSE) was used to deal with the class imbalance of the outcome variables as seen in Table 1 (3.3% major bleed, 2.6% stroke, 8.9% all-cause mortality). The R package used to implement the method was ‘ROSE’ (Lunardon, N., Menardi, G., & Torelli, N. (2014). ROSE: a package for binary imbalanced learning. R journal, 6(1).) The ROSE methodology was designed for classification tasks. It over-samples the minority class by generating synthetic examples derived from randomly selecting and adding perturbations to balance the class distribution.

**Material S4.**

Machine learning models have various tuneable parameters. Within this analysis, the default for all the models provided by the specified packages were used to aid in future replication. The models tested came from the ‘caret’ package and included: ‘glm’, ‘rf’, ‘lda’, ‘rpart’, ‘naïve-bayes’, ‘xgbTree’, and ‘nnet’. The full list of available models is provided here https://topepo.github.io/caret/available-models.html. Hence no parameter tuning was performed. However, within the logistic regression model, all available variables were incorporated, and interactions were permitted between variables.

**Material S5.**

When changing the threshold for CHADS_2_ to 3 and CHA_2_DS_2_-VASc to 3 in the 1-year application, the precision value of correct positive predictions had a minuscule increase (CHADS_2_ from 0.013 to 0.019; CHA_2_DS_2_-VASc from 0.011 to 0.012) but this was at the cost of a large decrease in the sensitivity of the model (CHADS_2_ from 0.761 to 0.470; CHA_2_DS_2_-VASc from 0.948 to 0.791). Hence, a justification of the set threshold levels has been demonstrated there was no additional benefit in altering them. A similar trend was observed when altering the risk categorisation threshold of the HAS-BLED score from 3 to 4 with the 1-year precision barely rising from 0.031 to 0.032 but a large reduction in sensitivity occurring, dropping from 0.155 to 0.021, confirming that the prediction rates were not enhanced by altering the threshold of these clinical risk scores.

**Material S6.**

All clinical decisions have different criteria of what is acceptable based on the specific situation. Given the absence of prediction tools with 100% accuracy, when developing models either sensitivity or specificity will be favoured. Clinically one of the main purposes of the current classification scheme with CHA_2_DS_2_-VASc is to initially identify patients who are truly low risk. Our analysis includes specificity to capture the occurrence of false positives (those with less risk) which could potentially result in unnecessary intervention or treatment. When assessing a patient for conditions such as cancer, typically the application will yield a higher sensitivity in order to catch more cases. However, when treatments or confirmatory testing is expensive, invasive or has possible side effects, typically specificity of the approach will be maximised. Within this paper, the metrics were balanced (Table 3), then both were separately optimised (Table 4).

**Material S7­­**

|  | **‘Stroke’** | | | | **‘No Stroke’** | | |
| --- | --- | --- | --- | --- | --- | --- | --- |
| **Baseline Characteristics** | | **Total Cohort (% ‘Stroke’ population)** | **Mean (SD)** | **Missing (% ‘Stroke’ population)** | **Total Cohort (% ‘No Stroke’ population)** | **Mean (SD)** | **Missing (% ‘No Stroke’ population)** |
| Age (years)  18-44  45-54  55-64  65-74  75-90 | 1 (0.1)  16 (2.3)  84 (12.3)  218 (32.0)  336 (49.3) | |  | 26 (3.8) | 422 (1.7)  1533 (6)  4685 (18.4)  9267 (36.3)  9205 (36.1) |  | 390 (1.5) |
| Gender  Female  Male | 319 (46.8)  362 (53.2) | |  | 0 (0.0) | 11414 (44.8)  14088 (55.2) |  | 0 (0.0) |
| Heart Rate |  | | 79.98 (20.8) | 9 (1.3) |  | 80.13 (21.3) | 201 (0.8) |
| Systolic Blood Pressure |  | | 135.1 (18.9) | 8 (1.2) |  | 132.3 (18.7) | 188 (0.7) |
| Diastolic Blood Pressure |  | | 77.98 (12.5) | 8 (1.2 |  | 78.05 (12.0) | 191 (0.7) |
| Height (cm) |  | | 166.6 (10.4) | 11 (1.6) |  | 168.22 (10.3) | 254 (1.0) |
| Weight (kg) |  | | 76.47 (19.0) | 11 (1.6) |  | 81.45 (20.6) | 208 (8.2) |
| BMI |  | | 27.43 (5.7) | 12 (1.8) |  | 28.66 (6.3) | 273 (1.1) |
| Treatment Group  Apixaban  ASA  Dabigatran  Edoxaban  Rivaroxaban  VKA  Antiplatelets other than ASA  None | 121 (17.8)  93 (13.7)  171 (25.1)  9 (1.3)  97 (14.2)  133 (19.5)  9 (1.3)  48 (7.0) | |  | 0 (0.0) | 4505 (17.7)  2163 (8.5)  8722 (34.2)  323 (1.3)  4015 (15.7)  4836 (19.0)  204 (0.8)  1338 (5.2) |  | 11 (1.6) |
| Region  Africa/Middle East  Asia  Europe  Latin America  North America | 3 (0.4)  178 (26.1)  322 (47.3)  40 (5.9)  138 (20.3) | |  | 0 (0.0) | 311 (1.2)  4727 (18.5)  12671 (49.7)  1967 (7.7)  5826 (22.8) |  | 0 (0.0) |
| Race  Arab/Middle East  Asian  Black/Afro-Caribbean  White  Other | 4 (0.6)  175 (25.7)  17 (2.5)  420 (61.7)  20 (2.9) | |  | 45 (6.6) | 327 (1.3)  4382 (17.2)  436 (1.7)  17780 (69.7)  875 (3.4) |  | 1702 (6.7) |
| Alcohol Use  No Alcohol  Less than 1 drink/week  1-7 drinks/week  More than 7 drinks/week | 324 (47.6)  156 (22.9)  115 (16.9)  44 (6.5) | |  | 42 (6.2) | 11087 (43.5)  6156 (24.1)  4979 (19.5)  1726 (6.8) |  | 1554 (6.1) |
| Smoking Status  Never Smoked  Current Smoker  Ex-Smoker | 370 (54.3)  84 (12.3)  214 (31.4) | |  | 13 (1.9) | 14785 (58.0)  2359 (9.3)  7536 (29.6) |  | 822 (3.2) |
| Types of AF |  | |  | 0 (0.0) |  |  | 0 (0.0) |
| Paroxysmal | 339 (49.8) | |  |  | 14195 (55.6) |  |  |
| Persistent | 260 (38.2) | |  |  | 8740 (34.3) |  |  |
| Permanent | 82 (12.0) | |  |  | 2567 (10.1) |  |  |
| Hypertension | 540 (79.3) | |  | 1 (0.1) | 19131 (75.0) |  | 55 (0.2) |
| Diabetes Mellitus | 181 (26.6) | |  | 0 (0.0) | 5886 (23.1) |  | 0 (0.0) |
| Coronary Artery Disease | 152 (22.3) | |  | 19 (2.3) | 4780 (18.7) |  | 643 (2.5) |
| Peripheral Artery Disease | 25 (3.7) | |  | 1 (0.1) | 731 (2.9) |  | 195 (7.6) |
| Previous Thromboembolism | 8 (1.2) | |  | 3 (0.4) | 3694 (14.5) |  | 0 (0.0) |
| Previous Myocardial Infarction | 71 (10.4) | |  | 1 (0.1) | 2421 (9.5) |  | 17 (0.07) |
| Previous Deep Vein Thrombosis | 8 (1.2) | |  | 3 (0.4) | 295 (1.2) |  | 312 (1.2) |
| Complex Aortic Plaque | 5 (0.7) | |  | 11 (1.6) | 250 (1.0) |  | 513 (2.0) |
| Respiratory Disease | 84 (12.3) | |  | 1 (0.1) | 2839 (11.1) |  | 269 (1.1) |
| **Outcomes** |  | |  |  |  |  |  |
| Major Bleed | 181 (26.6) | |  | 0 (0.0) | 692 (2.7) |  | 0 (0.0) |
| Stroke | 681 (100.0) | |  | 0 (0.0) | 0 (0.0) |  | 0 (0.0) |
| All-Cause Death | 258 (37.9) | |  | 0 (0.0) | 2070 (8.1) |  | 0 (0.0) |

Table S1: Characteristics of those who had an outcome of stroke and those did not have a stroke.

**Material S8**

|  | **‘Major Bleed’** | | | | **‘No Major Bleed’** | | |
| --- | --- | --- | --- | --- | --- | --- | --- |
| **Baseline Characteristics** | | **Total Cohort (% ‘Major Bleed’ population)** | **Mean (SD)** | **Missing (% ‘Major Bleed’ population)** | **Total Cohort (% ‘No Major Bleed’ population)** | **Mean (SD)** | **Missing (% ‘No Major Bleed’ population)** |
| Age (years)  18-44  45-54  55-64  65-74  75-90 | 2 (0.2)  19 (2.2)  99 (11.3)  291 (33.3)  433 (49.6) | |  | 0 (0.0) | 421 (1.7)  1530 (6.0)  4670 (18.5)  9194 (36.3)  9108 (36.0) |  | 0 (0.0) |
| Gender  Female  Male | 405 (46.4)  468 (53.6) | |  | 0 (0.0) | 11328 (44.8)  13928 (55.0) |  | 0 (0.0) |
| Heart Rate |  | | 79.6 (20.9) | 7 (0.8) |  | 80.2 (21.3) | 203 (0.8) |
| Systolic Blood Pressure |  | | 131.9 (19.5) | 5 (0.6) |  | 132.4 (18.7) | 191 (0.8) |
| Diastolic Blood Pressure |  | | 76.2 (12.2) | 5 (0.6) |  | 78.1 (12.0) | 194 (0.8) |
| Height (cm) |  | | 167.5 (10.3) | 7 (0.8) |  | 168.2 (10.3) | 258 (1.0) |
| Weight (kg) |  | | 81.2 (20.5) | 7 (0.8) |  | 81.3 (20.6) | 212 (0.8) |
| BMI |  | | 28.8 (6.5) | 9 (1.0) |  | 28.6 (6.3) | 276 (1.1) |
| Treatment Group  Apixaban  ASA  Dabigatran  Edoxaban  Rivaroxaban  VKA  Antiplatelets other than ASA  None | 159 (18.2)  53 (6.1)  207 (23.7)  10 (1.1)  169 (19.4)  230 (26.3)  5 (0.6)  40 (4.6) | |  | 0 (0.0) | 4346 (17.2)  2110 (8.3)  8515 (33.6)  322 (1.3)  3846 (15.2)  4606 (18.2)  208 (0.8)  1346 (5.3) |  | 11 (0.04) |
| Region  Africa/Middle East  Asia  Europe  Latin America  North America | 2 (0.2)  97 (11.1)  433 (49.6)  39 (4.5)  302 (34.6) | |  | 0 (0.0) | 312 (1.2)  4808 (19.0)  12560 (49.6)  1968 (7.8)  5662 (22.4) |  | 0 (0.0) |
| Race  Arab/Middle East  Asian  Black/Afro-Caribbean  White  Other | 4 (0.5)  103 (11.8)  29 (3.3)  668 (76.5)  14 (1.6) | |  | 55 (6.3) | 327 (1.3)  4454 (17.6)  424 (1.7)  17532 (69.3)  881 (3.5) |  | 1692 (6.7) |
| Alcohol Use  No Alcohol  Less than 1 drink/week  1-7 drinks/week  More than 7 drinks/week | 388 (44.4)  215 (24.6)  160 (18.3)  71 (8.1) | |  | 39 (4.5) | 11023 (43.6)  6097 (24.1)  4934 (19.5)  1699 (6.7) |  | 1557 (6.2) |
| Smoking Status  Never Smoked  Current Smoker  Ex-Smoker | 427 (48.9)  94 (10.8)  332 (38.0) | |  | 20 (2.3) | 14728 (58.2)  2349 (9.3)  7418 (29.3) |  | 815 (3.2) |
| Types of AF |  | |  | 0 (0.0) |  |  | 0 (0.0) |
| Paroxysmal | 447 (51.2) | |  |  | 14087 (55.7) |  |  |
| Persistent | 313 (35.9) | |  |  | 8687 (34.3) |  |  |
| Permanent | 113 (12.9) | |  |  | 2536 (10.0) |  |  |
| Hypertension | 706 (80.9) | |  | 2 (0.2) | 18965 (74.9) |  | 54 (0.2) |
| Diabetes Mellitus | 255 (29.2) | |  | 0 (0.0) | 5812 (23.0) |  | 0 (0.0) |
| Coronary Artery Disease | 218 (25.0) | |  | 26 (3.0) | 4714 (18.6) |  | 636 (2.5) |
| Peripheral Artery Disease | 44 (5.0) | |  | 12 (1.4) | 712 (2.8) |  | 184 (0.7) |
| Previous Thromboembolism | 150 (17.2) | |  | 0 (0.0) | 3739 (14.8) |  | 0 (0.0) |
| Previous Myocardial Infarction | 121 (13.9) | |  | 3 (3.4) | 2371 (9.4) |  | 15 (0.06) |
| Previous Deep Vein Thrombosis | 11 (1.3) | |  | 12 (1.4) | 292 (1.2) |  | 305 (1.2) |
| Complex Aortic Plaque | 11 (1.3) | |  | 27 (3.1) | 244 (1.0) |  | 497 (2.0) |
| Respiratory Disease | 147 (16.8) | |  | 8 (0.9) | 2776 (11.0) |  | 262 (1.0) |
| **Outcomes** |  | |  |  |  |  |  |
| Major Bleed | 873 (100.0) | |  | 0 (0.0) | 0 (0.0) |  | 0 (0.0) |
| Stroke | 181 (20.1) | |  | 0 (0.0) | 500 (2.0) |  | 0 (0.0) |
| All-Cause Death | 258 (29.6) | |  | 0 (0.0) | 2070 (8.2) |  | 0 (0.0) |

Table S2: Characteristics of those who had an outcome of major bleeding and those did not have a major bleed.

**GLORIA-AF Investigators**

Dzifa Wosornu Abban

Nasser Abdul

Atilio Marcelo Abud

Fran Adams

Srinivas Addala

Pedro Adragão

Walter Ageno

Rajesh Aggarwal

Sergio Agosti

Piergiuseppe Agostoni

Francisco Aguilar

Julio Aguilar Linares

Luis Aguinaga

Jameel Ahmed

Allessandro Aiello

Paul Ainsworth

Jorge Roberto Aiub

Raed Al-Dallow

Lisa Alderson

Jorge Antonio Aldrete Velasco

Dimitrios Alexopoulos

Fernando Alfonso Manterola

Pareed Aliyar

David Alonso

Fernando Augusto Alves da Costa

José Amado

Walid Amara

Mathieu Amelot

Nima Amjadi

Fabrizio Ammirati

Marianna Andrade

Nabil Andrawis

Giorgio Annoni

Gerardo Ansalone

M.Kevin Ariani

Juan Carlos Arias

Sébastien Armero

Chander Arora

Muhammad Shakil Aslam

M. Asselman

Philippe Audouin

Charles Augenbraun

S. Aydin

Ivaneta Ayryanova

Emad Aziz

Luciano Marcelo Backes

E. Badings

Ermentina Bagni

Seth H. Baker

Richard Bala

Antonio Baldi

Shigenobu Bando

Subhash Banerjee

Alan Bank

Gonzalo Barón Esquivias

Craig Barr

Maria Bartlett

Vanja Basic Kes

Giovanni Baula

Steffen Behrens

Alan Bell

Raffaella Benedetti

Juan Benezet Mazuecos

Bouziane Benhalima

Jutta Bergler-Klein

Jean-Baptiste Berneau

Richard A. Bernstein

Percy Berrospi

Sergio Berti

Andrea Berz

Elizabeth Best

Paulo Bettencourt

Robert Betzu

Ravi Bhagwat

Luna Bhatta

Francesco Biscione

Giovanni Bisignani

Toby Black

Michael J. Bloch

Stephen Bloom

Edwin Blumberg

Mario Bo

Ellen Bøhmer

Andreas Bollmann

Maria Grazia Bongiorni

Giuseppe Boriani

D.J. Boswijk

Jochen Bott

Edo Bottacchi

Marica Bracic Kalan

Drew Bradman

Donald Brautigam

Nicolas Breton

P.J.A.M. Brouwers

Kevin Browne

Jordi Bruguera Cortada

A. Bruni

Claude Brunschwig

Hervé Buathier

Aurélie Buhl

John Bullinga

Jose Walter Cabrera

Alberto Caccavo

Shanglang Cai

Sarah Caine

Leonardo Calò

Valeria Calvi

Mauricio Camarillo Sánchez

Rui Candeias

Vincenzo Capuano

Alessandro Capucci

Ronald Caputo

Tatiana Cárdenas Rizo

Francisco Cardona

Francisco Carlos da Costa Darrieux

Yan Carlos Duarte Vera

Antonio Carolei

Susana Carreño

Paula Carvalho

Susanna Cary

Gavino Casu

Claudio Cavallini

Guillaume Cayla

Aldo Celentano

Tae-Joon Cha

Kwang Soo Cha

Jei Keon Chae

Kathrine Chalamidas

Krishnan Challappa

Sunil Prakash Chand

Harinath Chandrashekar

Ludovic Chartier

Kausik Chatterjee

Carlos Antero Chavez Ayala

Aamir Cheema

Amjad Cheema

Lin Chen

Shih-Ann Chen

Jyh Hong Chen

Fu-Tien Chiang

Francesco Chiarella

Lin Chih-Chan

Yong Keun Cho

Jong-Il Choi

Dong Ju Choi

Guy Chouinard

Danny Hoi-Fan Chow

Dimitrios Chrysos

Galina Chumakova

Eduardo Julián José Roberto Chuquiure Valenzuela

Nicoleta Cindea Nica

David J. Cislowski

Anthony Clay

Piers Clifford

Andrew Cohen

Michael Cohen

Serge Cohen

Furio Colivicchi

Ronan Collins

Paolo Colonna

Steve Compton

Derek Connolly

Alberto Conti

Gabriel Contreras Buenostro

Gregg Coodley

Martin Cooper

Julian Coronel

Giovanni Corso

Juan Cosín Sales

Yves Cottin

John Covalesky

Aurel Cracan

Filippo Crea

Peter Crean

James Crenshaw

Tina Cullen

Harald Darius

Patrick Dary

Olivier Dascotte

Ira Dauber

Vicente Davalos

Ruth Davies

Gershan Davis

Jean-Marc Davy

Mark Dayer

Marzia De Biasio

Silvana De Bonis

Raffaele De Caterina

Teresiano De Franceschi

J.R. de Groot

José De Horta

Axel De La Briolle

Gilberto de la Pena Topete

Angelo Amato Vicenzo de Paola

Weimar de Souza

A. de Veer

Luc De Wolf

Eric Decoulx

Sasalu Deepak

Pascal Defaye

Freddy Del-Carpio Munoz

Diana Delic Brkljacic

N. Joseph Deumite

Silvia Di Legge

Igor Diemberger

Denise Dietz

Pedro Dionísio

Qiang Dong

Fabio Rossi dos Santos

Elena Dotcheva

Rami Doukky

Anthony D'Souza

Simon Dubrey

Xavier Ducrocq

Dmitry Dupljakov

Mauricio Duque

Dipankar Dutta

Nathalie Duvilla

A. Duygun

Rainer Dziewas

Charles B. Eaton

William Eaves

L.A Ebels-Tuinbeek

Clifford Ehrlich

Sabine Eichinger-Hasenauer

Steven J. Eisenberg

Adnan El Jabali

Mahfouz El Shahawy

Mauro Esteves Hernandes

Ana Etxeberria Izal

Rudolph Evonich III

Oksana Evseeva

Andrey Ezhov

Raed Fahmy

Quan Fang

Ramin Farsad

Laurent Fauchier

Stefano Favale

Maxime Fayard

Jose Luis Fedele

Francesco Fedele

Olga Fedorishina

Steven R. Fera

Luis Gustavo Gomes Ferreira

Jorge Ferreira

Claudio Ferri

Anna Ferrier

Hugo Ferro

Alexandra Finsen

Brian First

Stuart Fischer

Catarina Fonseca

Luísa Fonseca Almeida

Steven Forman

Brad Frandsen

William French

Keith Friedman

Athena Friese

Ana Gabriela Fruntelata

Shigeru Fujii

Stefano Fumagalli

Marta Fundamenski

Yutaka Furukawa

Matthias Gabelmann

Nashwa Gabra

Niels Gadsbøll

Michel Galinier

Anders Gammelgaard

Priya Ganeshkumar

Christopher Gans

Antonio Garcia Quintana

Olivier Gartenlaub

Achille Gaspardone

Conrad Genz

Frédéric Georger

Jean-Louis Georges

Steven Georgeson

Evaldas Giedrimas

Mariusz Gierba

Ignacio Gil Ortega

Eve Gillespie

Alberto Giniger

Michael C. Giudici

Alexandros Gkotsis

Taya V. Glotzer

Joachim Gmehling

Jacek Gniot

Peter Goethals

Seth Goldbarg

Ronald Goldberg

Britta Goldmann

Sergey Golitsyn

Silvia Gómez

Juan Gomez Mesa

Vicente Bertomeu Gonzalez

Jesus Antonio Gonzalez Hermosillo

Víctor Manuel González López

Hervé Gorka

Charles Gornick

Diana Gorog

Venkat Gottipaty

Pascal Goube

Ioannis Goudevenos

Brett Graham

G. Stephen Greer

Uwe Gremmler

Paul G. Grena

Martin Grond

Edoardo Gronda

Gerian Grönefeld

Xiang Gu

Ivett Guadalupe Torres Torres

Gabriele Guardigli

Carolina Guevara

Alexandre Guignier

Michele Gulizia

Michael Gumbley

Albrecht Günther

Andrew Ha

Georgios Hahalis

Joseph Hakas

Christian Hall

Bing Han

Seongwook Han

Joe Hargrove

David Hargroves

Kenneth B. Harris

Tetsuya Haruna

Emil Hayek

Jeff Healey

Steven Hearne

Michael Heffernan

Geir Heggelund

J.A. Heijmeriks

Maarten Hemels

I. Hendriks

Sam Henein

Sung-Ho Her

Paul Hermany

Jorge Eduardo Hernández Del Río

Yorihiko Higashino

Michael Hill

Tetsuo Hisadome

Eiji Hishida

Etienne Hoffer

Matthew Hoghton

Kui Hong

Suk keun Hong

Stevie Horbach

Masataka Horiuchi

Yinglong Hou

Jeff Hsing

Chi-Hung Huang

David Huckins

kathy Hughes

A. Huizinga

E.L. Hulsman

Kuo-Chun Hung

Gyo-Seung Hwang

Margaret Ikpoh

Davide Imberti

Hüseyin Ince

Ciro Indolfi

Shujiro Inoue

Didier Irles

Harukazu Iseki

C. Noah Israel

Bruce Iteld

Venkat Iyer

Ewart Jackson-Voyzey

Naseem Jaffrani

Frank Jäger

Martin James

Sung-Won Jang

Nicolas Jaramillo

Nabil Jarmukli

Robert J. Jeanfreau

Ronald D. Jenkins

Carlos Jerjes Sánchez

Javier Jimenez

Robert Jobe

Tomas Joen-Jakobsen

Nicholas Jones

Jose Carlos Moura Jorge

Bernard Jouve

Byung Chun Jung

Kyung Tae Jung

Werner Jung

Mikhail Kachkovskiy

Krystallenia Kafkala

Larisa Kalinina

Bernd Kallmünzer

Farzan Kamali

Takehiro Kamo

Priit Kampus

Hisham Kashou

Andreas Kastrup

Apostolos Katsivas

Elizabeth Kaufman

Kazuya Kawai

Kenji Kawajiri

John F. Kazmierski

P Keeling

José Francisco Kerr Saraiva

Galina Ketova

AJIT Singh Khaira

Aleksey Khripun

Doo-Il Kim

Young Hoon Kim

Nam Ho Kim

Dae Kyeong Kim

Jeong Su Kim

June Soo Kim

Ki Seok Kim

Jin bae Kim

Elena Kinova

Alexander Klein

James J. Kmetzo

G. Larsen Kneller

Aleksandar Knezevic

Su Mei Angela Koh

Shunichi Koide

Athanasios Kollias

J.A. Kooistra

Jay Koons

Martin Koschutnik

William J. Kostis

Dragan Kovacic

Jacek Kowalczyk

Natalya Koziolova

Peter Kraft

Johannes A. Kragten

Mori Krantz

Lars Krause

B.J. Krenning

F. Krikke

Z. Kromhout

Waldemar Krysiak

Priya Kumar

Thomas Kümler

Malte Kuniss

Jen-Yuan Kuo

Achim Küppers

Karla Kurrelmeyer

Choong Hwan Kwak

Bénédicte Laboulle

Arthur Labovitz

Wen Ter Lai

Andy Lam

Yat Yin Lam

Fernando Lanas Zanetti

Charles Landau

Giancarlo Landini

Estêvão Lanna Figueiredo

Torben Larsen

Karine Lavandier

Jessica LeBlanc

Moon Hyoung Lee

Chang-Hoon Lee

John Lehman

Ana Leitão

Nicolas Lellouche

Malgorzata Lelonek

Radoslaw Lenarczyk

T. Lenderink

Salvador León González

Peter Leong-Sit

Matthias Leschke

Nicolas Ley

Zhanquan Li

Xiaodong Li

Weihua Li

Xiaoming Li

Christhoh Lichy

Ira Lieber

Ramon Horacio Limon Rodriguez

Hailong Lin

Gregory Y. H. Lip

Feng Liu

Hengliang Liu

Guillermo Llamas Esperon

Nassip Llerena Navarro

Eric Lo

Sergiy Lokshyn

Amador López

José Luís López-Sendón

Adalberto Menezes Lorga Filho

Richard S. Lorraine

Carlos Alberto Luengas

Robert Luke

Ming Luo

Steven Lupovitch

Philippe Lyrer

Changsheng Ma

Genshan Ma

Irene Madariaga

Koji Maeno

Dominique Magnin

Gustavo Maid

Sumeet K. Mainigi

Konstantinos Makaritsis

Rohit Malhotra

Rickey Manning

Athanasios Manolis

Helard Andres Manrique Hurtado

Ioannis Mantas

Fernando Manzur Jattin

Vicky Maqueda

Niccolo Marchionni

Francisco Marin Ortuno

Antonio Martín Santana

Jorge Martinez

Petra Maskova

Norberto Matadamas Hernandez

Katsuhiro Matsuda

Tillmann Maurer

Ciro Mauro

Erik May

Nolan Mayer

John McClure

Terry McCormack

William McGarity

Hugh McIntyre

Brent McLaurin

Feliz Alvaro Medina Palomino

Francesco Melandri

Hiroshi Meno

Dhananjai Menzies

Marco Mercader

Christian Meyer

Beat j. Meyer

Jacek Miarka

Frank Mibach

Dominik Michalski

Patrik Michel

Rami Mihail Chreih

Ghiath Mikdadi

Milan Mikus

Davor Milicic

Constantin Militaru

Sedi Minaie

Bogdan Minescu

Iveta Mintale

Tristan Mirault

Michael J. Mirro

Dinesh Mistry

Nicoleta Violeta Miu

Naomasa Miyamoto

Tiziano Moccetti

Akber Mohammed

Azlisham Mohd Nor

Michael Mollerus

Giulio Molon

Sergio Mondillo

Patrícia Moniz

Lluis Mont

Vicente Montagud

Oscar Montaña

Cristina Monti

Luciano Moretti

Kiyoo Mori

Andrew Moriarty

Jacek Morka

Luigi Moschini

Nikitas Moschos

Andreas Mügge

Thomas J. Mulhearn

Carmen Muresan

Michela Muriago

Wlodzimierz Musial

Carl W. Musser

Francesco Musumeci

Thuraia Nageh

Hidemitsu Nakagawa

Yuichiro Nakamura

Toru Nakayama

Gi-Byoung Nam

Michele Nanna

Indira Natarajan

Hemal M. Nayak

Stefan Naydenov

Jurica Nazlić

Alexandru Cristian Nechita

Libor Nechvatal

Sandra Adela Negron

James Neiman

Fernando Carvalho Neuenschwander

David Neves

Anna Neykova

Ricardo Nicolás Miguel

George Nijmeh

Alexey Nizov

Rodrigo Noronha Campos

Janko Nossan

Tatiana Novikova

Ewa Nowalany-Kozielska

Emmanuel Nsah

Juan Carlos Nunez Fragoso

Svetlana Nurgalieva

Dieter Nuyens

Ole Nyvad

Manuel Odin de Los Rios Ibarra

Philip O'Donnell

Martin O'Donnell

Seil Oh

Yong Seog Oh

Dongjin Oh

Gilles O'Hara

Kostas Oikonomou

Claudia Olivares

Richard Oliver

Rafael Olvera Ruiz

Christoforos Olympios

Anna omaszuk-Kazberuk

Joaquín Osca Asensi

eena Padayattil jose

Francisco Gerardo Padilla Padilla

Victoria Padilla Rios

Giuseppe Pajes

A. Shekhar Pandey

Gaetano Paparella

F Paris

Hyung Wook Park

Jong Sung Park

Fragkiskos Parthenakis

Enrico Passamonti

Rajesh J. Patel

Jaydutt Patel

Mehool Patel

Janice Patrick

Ricardo Pavón Jimenez

Analía Paz

Vittorio Pengo

William Pentz

Beatriz Pérez

Alma Minerva Pérez Ríos

Alejandro Pérez-Cabezas

Richard Perlman

Viktor Persic

Francesco Perticone

Terri K. Peters

Sanjiv Petkar

Luis Felipe Pezo

Christian Pflücke

David N. Pham

Roland T. Phillips

Stephen Phlaum

Denis Pieters

Julien Pineau

Arnold Pinter

Fausto Pinto

R. Pisters

Nediljko Pivac

Darko Pocanic

Cristian Podoleanu

Alessandro Politano

Zdravka Poljakovic

Stewart Pollock

Jose Polo Garcéa

Holger Poppert

Maurizio Porcu

Antonio Pose Reino

Neeraj Prasad

Dalton Bertolim Précoma

Alessandro Prelle

John Prodafikas

Konstantin Protasov

Maurice Pye

Zhaohui Qiu

Jean-Michel Quedillac

Dimitar Raev

Carlos Antonio Raffo Grado

Sidiqullah Rahimi

Arturo Raisaro

Bhola Rama

Ricardo Ramos

Maria Ranieri

Nuno Raposo

Eric Rashba

Ursula Rauch-Kroehnert

Ramakota Reddy

Giulia Renda

Shabbir Reza

Luigi Ria

Dimitrios Richter

Hans Rickli

Werner Rieker

Tomas Ripolil Vera

Luiz Eduardo Ritt

Douglas Roberts

Ignacio Rodriguez Briones

Aldo Edwin Rodriguez Escudero

Carlos Rodríguez Pascual

Mark Roman

Francesco Romeo

E. Ronner

Jean-Francois Roux

Nadezda Rozkova

Miroslav Rubacek

Frank Rubalcava

Andrea M. Russo

Matthieu Pierre Rutgers

Karin Rybak

Samir Said

Tamotsu Sakamoto

Abraham Salacata

Adrien Salem

Rafael Salguero Bodes

Marco A. Saltzman

Alessandro Salvioni

Gregorio Sanchez Vallejo

Marcelo Sanmartín Fernández

Wladmir Faustino Saporito

Kesari Sarikonda

Taishi Sasaoka

Hamdi Sati

Irina Savelieva

Pierre-Jean Scala

Peter Schellinger

Carlos Scherr

Lisa Schmitz

Karl-Heinz Schmitz

Bettina Schmitz

Teresa Schnabel

Steffen Schnupp

Peter Schoeniger

Norbert Schön

Peter Schwimmbeck

Clare Seamark

Greg Searles

Karl-Heinz Seidl

Barry Seidman

Jaroslaw Sek

Lakshmanan Sekaran

Carlo Serrati

Neerav Shah

Vinay Shah

Anil Shah

Shujahat Shah

Vijay Kumar Sharma

Louise Shaw

Khalid H. Sheikh

Naruhito Shimizu

Hideki Shimomura

Dong-Gu Shin

Eun-Seok Shin

Junya Shite

Gerolamo Sibilio

Frank Silver

Iveta Sime

Tim A. Simmers

Narendra Singh

Peter Siostrzonek

Didier Smadja

David W. Smith

Marcelo Snitman

Dario Sobral Filho

Hassan Soda

Carl Sofley

Adam Sokal

Yannie Soo Oi Yan

Rodolfo Sotolongo

Olga Ferreira de Souza

Jon Arne Sparby

Jindrich Spinar

David Sprigings

Alex C. Spyropoulos

Dimitrios Stakos

Clemens Steinwender

Georgios Stergiou

Ian Stiell

Marcus Stoddard

Anastas Stoikov

Witold Streb

Ioannis Styliadis

Guohai Su

Xi Su

Wanda Sudnik

Kai Sukles

Xiaofei Sun

H. Swart

Janko Szavits-Nossan

Jens Taggeselle

Yuichiro Takagi

Amrit Pal Singh Takhar

Angelika Tamm

Katsumi Tanaka

Tanyanan Tanawuttiwat

Sherman Tang

Aylmer Tang

Giovanni Tarsi

Tiziana Tassinari

Ashis Tayal

Muzahir Tayebjee

J.M. ten Berg

Dan Tesloianu

Salem H.K. The

Dierk Thomas

Serge Timsit

Tetsuya Tobaru

Andrzej R. Tomasik.

Mikhail Torosoff

Emmanuel Touze

Elina Trendafilova

W. Kevin Tsai

Hung Fat Tse

Hiroshi Tsutsui

Tian Ming Tu

Ype Tuininga

Minang Turakhia

Samir Turk

Wayne Turner

Arnljot Tveit

Richard Tytus

C Valadão

P.F.M.M. van Bergen

Philippe van de Borne

B.J. van den Berg

C van der Zwaan

M. Van Eck

Peter Vanacker

Dimo Vasilev

Vasileios Vasilikos

Maxim Vasilyev

Srikar Veerareddy

Mario Vega Miño

Asok Venkataraman

Paolo Verdecchia

Francesco Versaci

Ernst Günter Vester

Hubert Vial

Jason Victory

Alejandro Villamil

Marc Vincent

Anthony Vlastaris

Jürgen vom Dahl

Kishor Vora

Robert B. Vranian

Paul Wakefield

Ningfu Wang

Mingsheng Wang

Xinhua Wang

Feng Wang

Tian Wang

Alberta L. Warner

Kouki Watanabe

Jeanne Wei

Christian Weimar

Stanislav Weiner

Renate Weinrich

Ming-Shien Wen

Marcus Wiemer

Preben Wiggers

Andreas Wilke

David Williams

Marcus L. Williams

Bernhard Witzenbichler

Brian Wong

Ka Sing Lawrence Wong

Beata Wozakowska-Kaplon

Shulin Wu

Richard C. Wu

Silke Wunderlich

Nell Wyatt

John (Jack) Wylie

Yong Xu

Xiangdong Xu

Hiroki Yamanoue

Takeshi Yamashita

Ping Yen Bryan Yan

Tianlun Yang

Jing Yao

Kuo-Ho Yeh

Wei Hsian Yin

Yoto Yotov

Ralf Zahn

Stuart Zarich

Sergei Zenin

Elisabeth Louise Zeuthen

Huanyi Zhang

Donghui Zhang

Xingwei Zhang

Ping Zhang

Jun Zhang

Shui Ping Zhao

Yujie Zhao

Zhichen Zhao

Yang Zheng

Jing Zhou

Sergio Zimmermann

Andrea Zini

Steven Zizzo

Wenxia Zong

L Steven Zukerman
